# Development and Verification of a Risk Index for Evaluating the Chemical Accident Risk of Korean Chemical Enterprises

**DOI:** 10.3390/ijerph16224409

**Published:** 2019-11-11

**Authors:** Saemi Shin, Sang-Hoon Byeon, Jong-Ryeul Sohn, Kyong Whan Moon

**Affiliations:** School of Health and Environmental Science, Korea University, Seoul 02841, Korea; saemishin@naver.com (S.S.); sohn1956@korea.ac.kr (J.-R.S.); kwmoon@korea.ac.kr (K.W.M.)

**Keywords:** KCARI, chemical accident, risk index, relative ranking method, inherent safety

## Abstract

The scale of the damage due to chemical accidents in Korea is significant, and appropriate preparation and response are required. Currently, Korean enterprises are managed on the basis of the presence of certain substances. However, chemicals other than these also cause chemical accidents. It is necessary to develop a relative ranking risk index that can be calculated through use of the chemical enterprise information on chemical enterprises that is available. The Korean chemical accident risk index (KCARI), which consists of the flammability, reactivity, explosiveness, corrosiveness, toxicity, and inventory sub-indices, was developed and verified by determining the for difference in KCARI was performed by accident, and accident severity category, calculating the correlation between the KCARI values, the factors, and some sub-indices, determining how an increase in the KCARI would impact how the incident rate changed as KCARI increased and how well the KCARI can predict the chemical accident risk of chemical handling enterprises, and confirming the consistency of the proposed index and the current system. These results indicated that the frequency and severity of chemical accidents, and the presence of accidental substances, showed significant differences in the KCARI values. However, there were limitations in the ability of the fitted model to precisely predict the accident. Thus, this model can be used as a tool for the early screening and management of enterprises with a high risk of chemical accident.

## 1. Introduction

Approximately 40,000 chemical substances are used in Korea, and the number of chemical substances and their use are continuously increasing [1]. With the aging of chemical industrial complexes, concerns regarding chemical accidents have increased due to the occurrence of serious chemical accidents, such as the 1991 Nakdong River phenol contamination incident, 2005 hydrogen chloride leakage at Yeosu Industrial Complex, and 2008 phenol leakage in Gimcheonand [2]. The 2012 hydrogen fluoride leakage accident, which led to 23 casualties and 50 billion won of property damage, caused a significant increase in the public’s interest regarding chemicals and chemical accidents [3]. Accordingly, the Korea Ministry of Environment (MoE) has reformed the chemical management system by enacting and enforcing the Chemical Control Act (CCA) and the Act on Registration, Evaluation, etc. of Chemicals [4].

Article 10 of the CCA discusses a list of chemical substances, the volume of chemicals to be handled, and the history of chemical accidents. MoE collects this information for enterprises that handle chemicals in amounts of 100 kg/year or more, and the findings are disclosed to the public online. Under the CCA, approximately 45,000 chemicals and 16,000 enterprises are managed. The MoE also discloses details of workplace chemical accidents through their online system, Chemistry Safety Clearing-house (CSC).

According to the CSC statistics, there were 200 chemical accidents at 117 workplaces in Korea from 2012 to 2014 [5]. Furthermore, a total of 113 chemical accidents took place in 2015, and 78 and 87 chemical accidents took place in 2016 and 2017, respectively. 593 casualties occurred, including 25 deaths and 572 injured. Thus, the scale of the damage due to chemical accidents is significant, and appropriate preparation and response are required.

Currently, the MoE focuses on a hazard control program for the substances that require preparation for accidents. The intent of this is to prepare for, and respond to, chemical accidents. The substances requiring preparation for accidents are designated by Article 39 of the CCA. Moreover, Article 41 of the CCA requires a hazard control program for workplaces that contain a certain amount of substances requiring preparation for accidents. The hazard control program includes off-site consequence analysis (OCA). The MoE has the authority to judge conformity, demand corrective action, or impose penalties on the hazard control program.

According to Article 42 of the CCA, the enterprise shall notify the residents of the risk within a workplace once per year. Following the occurrence of an incident, the MoE provides information to the relevant agencies regarding the predicted damaged area and accident control through the private software, CARIS [6].

The currently revised Korean chemical accident preparation and response system has three problems. First, enterprises are managed on the basis of the presence of certain substances. Second, public, regular, or upright access routes are not provided to the local residents in the case of a chemical accident. Third, risk information is transferred to the relevant agencies in response to accidents after the occurrence of an incident.

Through “The Chemical Accident Prevention Technology Development Project” (2015–2020), the MoE is developing a new system that provides a risk map that is similar to CARIS to the relevant agencies in response to accidents before an incident. The precautionary risk of workplace chemical accidents is additional information that is provided beyond the CARIS system.

In the existing system, risk management of chemical accidents was confined to workplaces that have substances requiring preparation for accidents in accordance with the CCA. However, chemical accidents are also caused by chemicals other than these [7]. According to the CSC statistics from 2014 to 2017, in total, 384 chemical accidents occurred. 202 (52.6%) occurred in working processes and storage tanks, 171 (44.5%) occurred in businesses that manufacture, use, or keep or store chemicals. A large proportion of chemical accidents occur in chemical handling enterprises. Thus, in the new system, the risk evaluation of chemical accidents should be performed differently than the current hazard control program and it should be conducted throughout all chemical handling enterprises.

The MoE recommends using a layer of protection analysis as a risk assessment method for the enterprises that handle substances requiring preparation for accidents [8]. However, for enterprises that do not handle substances requiring preparation for accidents, information cannot be obtained to perform a quantitative or semi-quantitative risk assessment. Thus, a relative ranking method of calculating an index result is an appropriate means of risk assessment.

Several relative ranking methods have been developed, including Dow’s Fire and Explosion Index, Mond Fire, Explosion and Toxicity Index, Toxicity Hazard Index, Safety Weighted Hazard Index, and Inherent Safety Index (ISI) [9,10,11,12,13]. However, these methods still require too much information to be applied to enterprises that do not handle substances requiring preparation for accidents. Therefore, it is necessary to develop a relative ranking risk index that can be calculated through the use of the chemical enterprise information that is collected by Article 10 of the CCA.

Kletz [14] and Heikkila [15] have argued that the concept of inherent safety should be applied to process design or process route selection. Many inherent safety indices have been developed since the development of the Prototype Index of Inherent Safety of Edwards [16] and the ISI of Heikkilä [11] in the 1990s [17]. An inherently safe design avoids risk by reducing the amount of chemicals or the number of hazardous processes rather than managing risks. The ISI focuses on assessing the quantity and risks of the chemicals and the risks of the process itself rather than the changes in risks that are caused by the management of the process. Unlike the Dow index and the Mond index, which require a significant amount of detailed information, the ISI can be used in an early design stage, where much information is not disclosed, because it utilizes the natural process information [15].

The information that was collected by Article 10 of the CCA is a factor that determines the inherent safety of a workplace. Through the inherent safety index that was developed by combining the information collected by Article 10 of the CCA, it is possible to screen workplaces with a high risk of chemical accidents. In this study, we developed a risk index that can be used to calculate the risk of chemical accidents in the workplaces that handle chemicals. The index developed herein can be calculated through the publicly collected data in Korea. Additionally, the index can be verified by comparing and analyzing the risk index that is calculated by substituting the actual accident history data.

## 2. Materials and Methods

### 2.1. Proposal of the Chemical Accident Risk Index Model

The Korean chemical accident risk index (KCARI) is an index that is designed to predict risk. Risk is calculated as the product of consequence and frequency, as shown in Equation (1) [18].

Risk = Consequence × Frequency(1)

The index was calculated as a score rather than a ranking, category, or class in order to verify that the results calculated by the relative ranking method represent the components of the risk (consequence, frequency).

The KCARI consists of the following sub-indices: flammability, reactivity, explosiveness, corrosiveness, toxicity, and inventory. Other sub-indices are available for the individual chemicals; however, in the case of inventory, the storage volume is collected and released as the total amount of chemicals, according to Article 10 of the CCA.

The individual chemical factor (ICF), which is composed of flammability, reactivity, explosiveness, corrosiveness, and toxicity, is calculated for each chemical since the sub-indices have different dimensions. The KCARI is calculated by multiplying the maximum ICF value (mICF) by the plant factor (PF), which is composed of the handling volume, storage, and number of chemicals handled in a given workplace. Equation (2) shows this calculation.

KCARI = mICF × PF(2)

mICF = max sum (flammability, reactivity, explosiveness, corrosiveness, and toxicity sub-indices of each chemical); PF = sum (handling volume, storage, and number of chemicals sub-indices of plants)

The NFPA 704 code was used for the flammability and reactivity KCARI sub-indices, which the National Fire Protection Association (NFPA) provides as a standard [19]. In the case of explosiveness, the difference between the upper exposure limit and lower exposure limit was used to obtain a rating. The NFPA code and physical property information were obtained from the comprehensive chemical information system that is operated by the MoE. For corrosiveness and toxicity, the Global Harmonized System (GHS) classification was used for rating. Toxicity is limited to toxic categories that have immediate effects upon contact or inhalation. For the GHS hazard classification, chemical information that was operated by Korea Occupational Safety and Health Agency was used, see Table A1 of Appendix A.

The amount of annual circulation of the chemicals was graded on the basis of figures that are meaningful under the CCA to facilitate the understanding and utilization of domestic stakeholders. According to Article 23 of the CCA, which is the OCA regulation, the classification levels of the annual chemical amounts are less than 100 tons/more than 100 tons and less than 1000 tons/more than 1000 tons. According to Article 23 of the CCA, the Hazardous Chemical Supervisor regulation, the classification levels of the annual chemical amounts are more than 1000 tons and less than 10,000 tons/more than 10,000 tons and less than 100,000 tons/more than 100,000 tons. We classify these grades into five total grades by integrating these two criteria, see Table A2 of Appendix A.

Similarly, the workplace storage was graded on the basis of figures that are meaningful under the CCA. Classification is made by applying less than 0.5 tons/more than five tons, less than five tons/more than five tons less than 50 tons/more than 50 tons, less than 500 tons/more than 500 tons, according to the quantity of 0.5 tons to which the legal regulation is imposed by Article 15 of the CCA, see Table A2 of Appendix A.

An example of the application of this technique is shown through Plant A. By Article 10 of the CCA, enterprises report the amount of chemical products handled and the proportion of each ingredient in the product to the government, and the government combines the data and releases the amount of ingredients. In Plant A with chemical storage volumes and chemical handling volume shown in Table 1, KCARI is calculated as the product of mICF and PF calculated through the scoring process shown in Table 2 and Table 3.

### 2.2. Calculation of Risk Index and Model Validation

A group of domestic workplaces were selected to calculate the risk index and to collect accident history information in order to verify the proposed model. In the case of the chemical information by enterprise, the information that is publicly available in accordance with Article 10 of the CCA was classified into categories. Thus, micro data were obtained from the MoE, and the raw value was used for calculating the risk index.

In this study, we focused on enhancing the internal validity of the verification process by limiting the region and businesses. According to the CSC statistics, Seoul and Gyeonggi province have the highest incidence rates among the regions, and businesses that manufacture and use chemicals have the highest incidence rates among businesses; therefore, these regions and businesses were selected as the focus of this study. The KCARI was calculated for 946 workplaces, where data on chemical handling were available, and the accident history was examined.

The KCARI was calculated for each workplace. The average and standard deviation were calculated, depending on whether accidents occurred or not, and each accident was categorized by its severity (no accident, accident with no casualties, and accident with casualties). The *t*-test was performed to determine the difference in KCARI by accident and by accident severity category.

By calculating the correlation between the KCARI values, the factor that was attributed to the plant and sub-indices, the factor attributed to the plant and its sub-indices, and the sub-indices of each factor, it can be determined how much the lower-tier components are related to the higher-tier components and whether a correlation between components of the same tier exists.

A binary logistic regression analysis was performed. Additionally, the model fit was validated to determine how an increase in the KCARI would impact the incident rate and determine how well the KCARI can predict the chemical accident risk of chemical handling enterprises. A multinomial logistic analysis was performed to observe the impact of the accident severity category as the KCARI increased. A discriminant analysis was performed, and the reclassification error rate was measured through the derived discriminant function in order to verify whether the category of accident severity can be predicted through the KCARI.

The difference of the KCARI was confirmed by a *t*-test, and the difference of the accident rate and the accident severity category was confirmed by chi-square test to confirm the consistency of the proposed index and the current system, which manages enterprises that handle a certain amount of substances requiring preparation for accidents.

## 3. Results

Among the 946 enterprises in Seoul and Gyeonggi province, 35 enterprises were found to have chemical accidents at least once, and 14 of these accidents had casualties.

**Figure 1:**
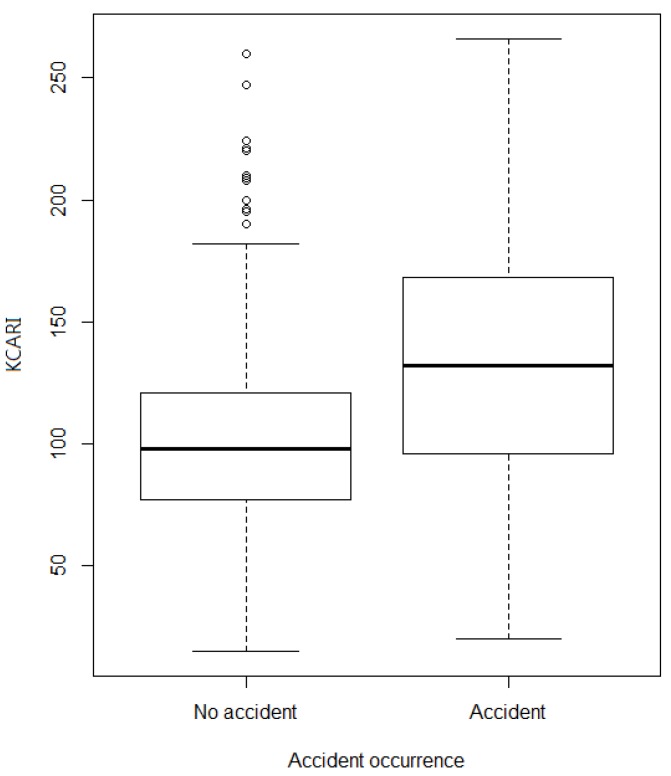
The average KCARI by accident occurrence.

**Figure 2:**
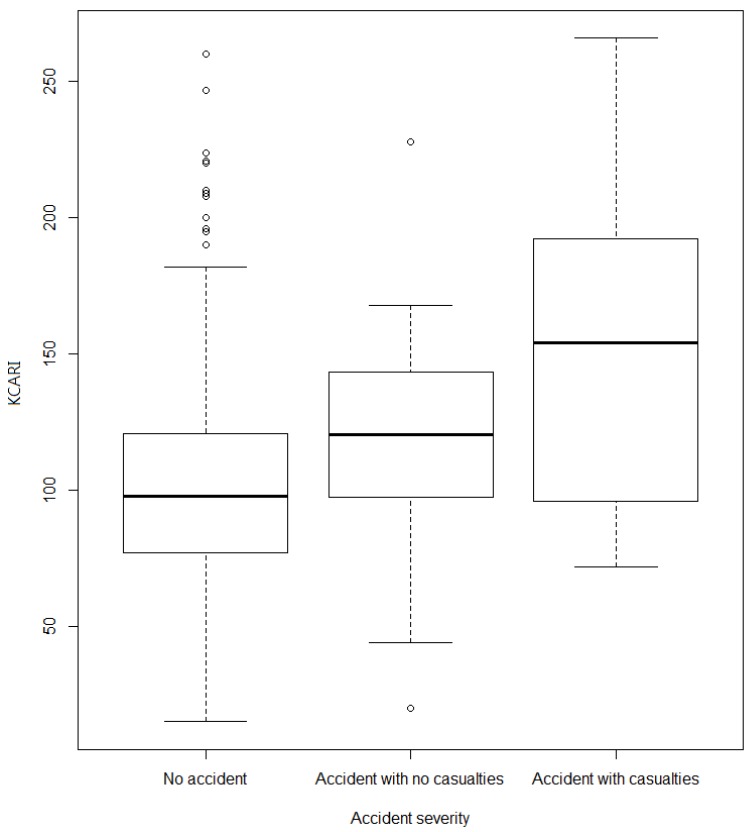
The average KCARI values by accident severity category.

The KCARI was found to be 103.7 ± 39.2 for all of the workplaces, 102.5 ± 38.1 in the workplaces without an accident history and 134.3 ± 54.1 in the workplaces with an accident history, and there was a significant difference according to the accident status (*p*-value = 0.001), as shown in Figure 1. The KCARI also had a significant difference according to the accident severity (*p*-value < 0.001), providing results of 119.8 ± 45.3 in accident workplaces without casualties and 153.7 ± 64.2 in accident workplaces with casualties, as shown in Figure 2.

**Figure 3:**
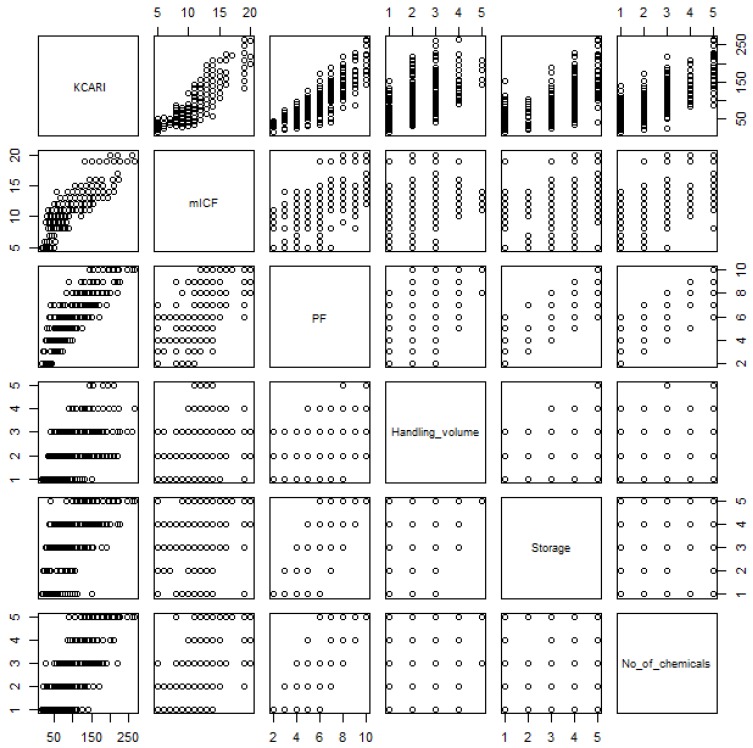
The scatter plot matrix of the index, factors, and PF sub-indices.

The results of the correlation analysis between the index, factors, and sub-indices that are attributed to the plant indicate the following: the correlation coefficients between KCARI, the two factors of KCARI, and the three sub-indices constituting PF were 0.58–0.90; the correlation coefficients between PF and the three sub-indices constituting PF were 0.40–0.85; the correlation coefficient between mICF and PF was 0.56; the correlation coefficient between the handling volume, storage, and number of chemicals was 0.26–0.47; the correlation coefficients between mICF and the three sub-indices constituting PF were 0.27–0.61. The variation inflation factor between the components of the same layer ranged from 1.22 to 1.41, and multicollinearity was not exhibited. The correlation coefficients between the components of the upper tier and those of the lower tier constituting the upper tier is low to very high, and the correlation coefficients between the same or the non-hierarchical elements are low to moderate [20] and they are relatively low as compared to the former. As a result, the relationships between the components appeared to be appropriate, as shown in Table 4 and Figure 3.

**Figure 4:**
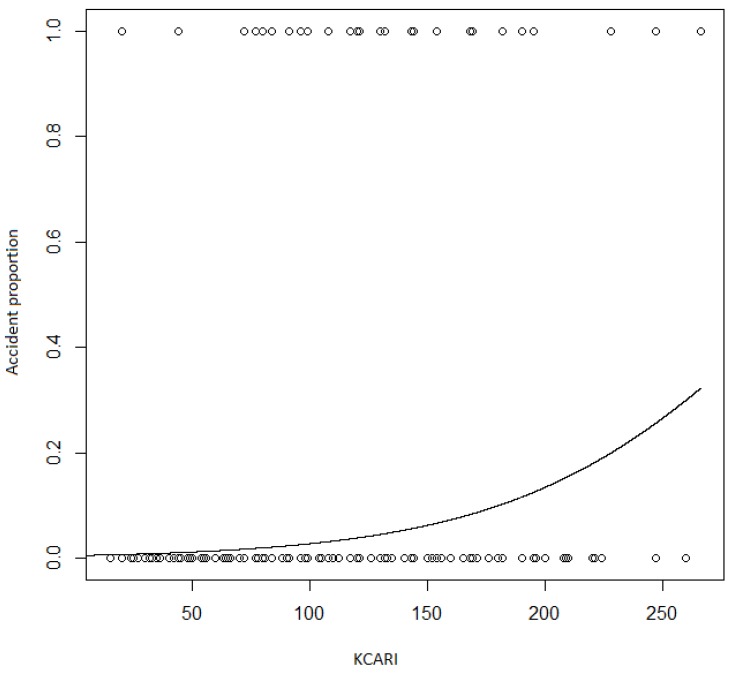
The accident proportion by KCARI.

The log odds of the accidents by the increase of KCARI were 0.017, but the coefficient of determination was 0.074 (*p*-value < 0.001), as shown in Figure 4. The log odds between no accidents and accidents with no casualties by the increase of KCARI was 0.010 (*p*-value < 0.001). Additionally, the log odds between no accidents and accidents with casualties by the increase of KCARI was 0.025 (*p*-value < 0.001), but the coefficient of determination was as low as 0.079.

**Figure 5:**
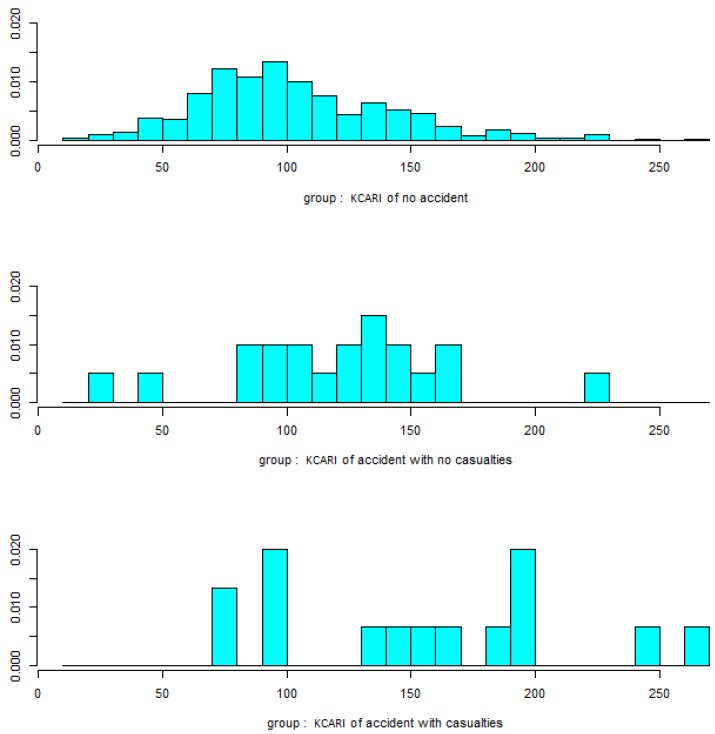
Discriminant histogram of KCARI by accident severity category.

As a result of the discriminant analysis, the coefficient of the linear discriminant function was 0.026, and, as shown in the results of the ANOVA, there was a significant difference between the groups. The reclassification error rate was 3.7%; however, only one of the actual accidents was predicted. As a result, accident prediction through the discriminant function was not successful. Figure 5 shows the KCARI linear histograms by accident severity category.

The results indicate a tendency to increase the incidence rate or accident severity according to KCARI; however, there was a limit to precisely predicting the accident through the fitted model. This is because the variance of the predictive variable within the result variable category was large and the explanatory power was low.

The KCARI was found to be 96.1 ± 39.3 for workplaces without substances and 128.2 ± 38.4 for workplaces with substances. The results show a significant difference in the KCARI based on whether substances are handled (*p*-value < 0.001). The difference in the accident incidence and accident severity category was found to be statistically significant (*p*-value = 0.001). However, 18 out of 35 cases of accidents and nine out of 14 cases of accidents with casualties were found in workplaces that do not handle substances, and the false negative rates were 51.4% and 64.3%, respectively. Thus, it has been found that there is a limit to screening workplaces that are at risk of accidents through the present system.

## 4. Discussion

We examined the relative ranking methods for inherent safety in the safety area to determine the accident risk, in the occupational health area to determine the risk of occupational diseases, and in the environmental area to judge the environmental impact due to the chemical leakages in order to develop this model. In many cases, the ISI [11] was used or ISI-based methodologies were strengthened [21,22,23,24]. This model is also based on the ISI but considers the range of data available in Korea and adjusts some factors. ISI consists of the sum of the Chemical Inherent Safety Index and the Process Inherent Safety Index, each of which is the sum of the sub-indices [15]. Relative ranking methods, such as ISI, are the techniques used to derive approximate risk levels from insufficient or uncertain information. The ISI on which the model is based and the relative ranking method referred to in model development are simple and conceptual calculations. According to this trend, this model also calculates the ICF and PF as the sum of the scores that are assigned by category for each sub-index.

The threshold limit value (TLV) or occupational exposure limit (OEL) is typically used as the toxicity indicator in relative ranking methods for assessing the inherent safety [11,21,25,26,27,28,29]. However, the TLV or OEL is established with consideration of technical, socioeconomic, and political issues [30,31], and it was decided that the herein method utilizes qualitative classification that only considers the hazards. Hazardous substances can be classified by the risk phrases of the European Union [27,32,33] or the health effect codes that are based on the OSHA guidelines that are presented in the field operation manual [26]. In this model, the GHS hazard classification and labeling of chemicals, which was converted from the risk phrase study that was presented by Hassim [27], was used. For corrosiveness, which has typically been used in the relative ranking method [27,29,34], the GHS classification of chemicals was used instead, because the material of the storage container in the workplace cannot be identified by the data that are available in Korea.

In the case of the ISI, the flammability sub-index was constructed whileusing the flash point and boiling point, and the reactivity sub-index was constructed using heat of main and side reaction. However, in this model, we refer to other literature to generate an easy-to-use index, and thus, NFPA F [12,35,36] and NFPA R [21] were used.

Although the concept of risk should be numerically defined for verification, it is impossible to quantitatively calculate the risk with insufficient information on the process factors whose frequency can be estimated. As shown in Equation (1), the concept of risk is separated into severity and frequency, which can be numerically explained and used for verification. The results show that the incidence rate or the severity of accidents increases with KCARI, but it has been confirmed that there is a limit in the ability of the model to accurately predict the accident. The KCARI is not inherently feasible to accurately predict risks, because the lack of data does not fairly reflect the activity or condition and management status of a process, and thus, the portions that the model cannot explain are likely to be due to these factors. Rather than using the model to accurately predict the risk of an accident, the model should be used to screen for hazardous workplaces among all sites, including those that are not covered by Article 41 of the CCA.

A lack of information also affected verification. We tried to compare the results of this study with the results of other methods and considered the application of the relative ranking technique for inherent safety that was developed previously in order to judge the adequacy of the model, but it was not possible to apply existing methods under the Korean chemical control system.

Most of inherent safety indexes examined required process information, and it is not possible to supply the information that is required by the index through the Korean chemical control system. One of the indices reviewed did not use process information [32], and some indices are composed of several factors or sub-indices that do not require process information and may use them separately [21,23,24,26,27,28,29]. In those cases, TLV [28], which is the sum of the scores of TLV and R-phrase [27], NFPA H, and OSHA HE [26], or the sum of the scores assigned to the flash point or boiling point, explosion limit, NFPA R, and TLV [21] was used. However, these cases were not applicable either, because the units of measurement for the indices are a chemical or a process, and the methods do not include a method for incorporating values that are calculated for different chemicals or processes into units of enterprises.

It is necessary to secure process information in order to overcome the accuracy limitations of the model. The MoE requires enterprises to submit information about their hazardous processes and the consequence area through the OCA, in accordance with Article 23 of the CCA. However, this information is not collected as data and is not transmitted to the stakeholders; it is only used as evidence to determine the suitability of the chemical handling facility. It will be necessary to collect and construct data that could be obtained through the OCA system to more accurately predict chemical accidents.

The verification of this model is limited to a specific region and industry for increasing and enhancing internal validity. However, this model is intended for all sites handling chemicals. The KCARI is developed as an index that can be calculated by utilizing the information that is collected by Article 10 of the CCA. Article 10 of the CCA regulates the collection of information at chemical handling sites. This provision applies to all workplaces that handle more than 100 kg/y of total chemicals. Hence, it is necessary to reevaluate the suitability of the model that is presented herein by expanding the study area and industry to nationwide and all industries, respectively. Evaluating the risks of the entire chemical handling site and using the results is only one activity of an experimental government project that currently has no legal basis. It is necessary to establish a legal institutional basis for the utilization plan in order to secure the effectiveness of this model. When institutionalization is carried out, consideration should be given to improving the accessibility of the public.

In several countries and regions, similar to this model, chemical risk assessments are being conducted based on the data available in their respective regions [37,38,39]. Thus, the form of the index is affected by the information that is available and the characteristics of the area. It would be possible to modify or reinforce indices with the availability of additional information due to legal and institutional maintenance or the characteristics and needs of local demand.

## 5. Conclusions

The purpose of this study is to overcome the limitations of the Korean accident preparation system, which is currently managed on the basis of specific chemicals. Thus, a risk index model, the KCARI, is constructed based on an ISI to screen for enterprises that are at risk of a chemical accident. The frequency and severity of chemical accidents and the presence of accidental substances showed significant differences in the KCARI values. The frequency and severity of accidents increased significantly with increasing KCARI at workplaces that manufacture or use chemicals in Seoul and Gyeonggi province. However, there were limitations in the ability of the fitted model to precisely predict an accident. This is because the variance of the predictive variable within the variable category of the result was large and the explanatory power was low. Thus, this model can be used as a tool for the early screening and management of enterprises with a high risk of chemical accident. In the future, research should be conducted to contribute to the preparation and risk management of chemical accidents by securing more information by including process information, expanding target areas and industries, and devising ways to systematically use this model.

## Figures and Tables

**Table 1:** Chemicals and their handling volume of example enterprise.

The Name of Enterprise	Industry	The Storage Volume of Plant (ton)	Chemical (CASRN)	The Handling Volume of Chemical (ton)
Plant A	Using	657.8	Nitric acid (7697-37-2)	148.5
			Hydrogen peroxide (7722-84-1)	565.75
			Hydrofluoric acid (7664-39-3)	240.9
			Sodium hydroxide (1310-73-2)	167.9
			Potassium hydroxide (1310-58-3)	1.8
			Antimony (III) oxide (1309-64-4)	0.001
			Ammonium hydroxide (1336-21-6)	480.8
			Hydrochloric Acid (7647-01-0)	98.5
			Phosphorus (V) oxychloride (10025-87-3)	0.09
			Ammonium fluoride (12125-01-8)	70.4
			Sulfuric acid (7664-93-9)	861.5

**Table 2:** The scoring to the individual chemical factor (ICF) of each chemical and maximum ICF value (mICF) of example enterprise.

Chemical	Flammability		Reactivity		Explosiveness		Corrosiveness		Toxicity (Highest Score Characteristic)		ICF	m-ICF
	NFPA F	Score	NFPA R	Score	UEL–LEL	Score	GHS *	Score	GHS *	Score		
Nitric acid	1	2	0	1	-	1	-	1	SC	5	10	12
Hydrogen peroxide	1	2	0	1	-	1	-	1	SC	5	10	
Hydrofluoric acid	1	2	0	1	-	1	C-1	2	AT-1, SC	5	11	
Sodium hydroxide	1	2	0	1	-	1	C-1	2	SC	5	11	
Potassium hydroxide	1	2	0	1	-	1	C-1	2	SC	5	11	
Antimony (III) oxide	0	1	0	1	-	1	-	1	-	1	5	
Ammonium hydroxide	0	1	0	1	-	1	-	2	SC	5	10	
Hydrochloric Acid	1	2	0	1	-	1	-	1	SC	5	10	
Phosphorus (V) oxychloride	2	3	0	1	-	1	-	1	AT-1, SC	5	11	
Ammonium fluoride	0	1	0	1	-	1	-	1	AT-3	4	8	
Sulfuric acid	2	3	0	1	-	1	C-1	2	AT-2, SC	5	12	

* Substance corrosive to metal: C; Skin corrosive: SC; Acute toxicity: AT; - category number

**Table 3:** The scoring to the plant factor (PF) of example enterprise.

Handling Volume		Storage		The Number of Chemicals		PF
Total Handling Volume of Chemicals (ton)	Score	The Storage Volume of Plant (ton)	Score	The Number of Chemicals	Score	
2636.141	3	657.8	5	11	5	13

**Table 4:** Correlation coefficients of the index, factors, and sub-indices of PF.

	KCARI	mICF	PF	Handling Volume	Storage	No. of Chemical
KCARI	1	-	-	-	-	-
mICF	0.77	1	-	-	-	-
PF	0.90	0.56	1	-	-	-
Handling volume	0.58	0.27	0.40	1	-	-
Storage	0.74	0.35	0.83	0.47	1	-
No. of chemical	0.79	0.61	0.85	0.26	0.46	1

## Appendix A

**Table A1:** Criteria and score of the individual chemical factor sub-indices.

Sub-index	Criteria	Score
Flammability	NFPA 704 Flammability rating = 0	1
	NFPA 704 Flammability rating = 1	2
	NFPA 704 Flammability rating = 2	3
	NFPA 704 Flammability rating = 3	4
	NFPA 704 Flammability rating = 4	5
Reactivity	NFPA 704 Reactivity rating = 0	1
	NFPA 704 Reactivity rating = 1	2
	NFPA 704 Reactivity rating = 2	3
	NFPA 704 Reactivity rating = 3	4
	NFPA 704 Reactivity rating = 4	5
Explosiveness	Not explosive	1
	0 ≤ Difference of Upper and Lower Explosive Limits < 20	2
	20 ≤ Difference of Upper and Lower Explosive Limits < 45	3
	45 ≤ Difference of Upper and Lower Explosive Limits < 70	4
	70 ≤ Difference of Upper and Lower Explosive Limits	5
**GHS Hazard Classification**		
Corrosiveness	Substances corrosive to metal—No category	1
	Substances corrosive to metal—Category 1	2
**GHS Hazard Classification**		
Toxicity	Other or no health hazard category	1
	Skin Irritation—Category 2; Eye Irritation—Category 2; Target organ systemic toxicity: single—Category 3	2
	Acute toxicity (dermal, inhalation)—Category 4; Aspiration Toxicity—Category 1–2	3
	Acute toxicity (dermal, inhalation)—Category 3; Serious Eye Damage—Category 1; Skin Sensitization—Category 1; Respiratory Sensitization—Category 1	4
	Acute toxicity (dermal, inhalation)—Category 1–2; Skin Corrosion—Category 1	5

**Table A2:** Criteria and score of the plant factor sub-indices.

Sub-index	Criteria	Score
Handling volume	<100 ton	1
	100 ton ≤, <1000 ton	2
	1000 ton ≤, <10,000 ton	3
	10,000 ton ≤, <100,000 ton	4
	100,000 ton≤	5
Storage	<0.5 ton	1
	0.5 ton ≤, <5 ton	2
	5 ton ≤, <50 ton	3
	50 ton ≤, <500 ton	4
	500 ton≤	5
No. of chemicals	1	1
	2–3	2
	4–9	3
	10–15	4
	16–	5
